# Quantitative and combinatory determination of *in situ* phosphorylation of tau and its FTDP-17 mutants

**DOI:** 10.1038/srep33479

**Published:** 2016-09-19

**Authors:** Taeko Kimura, Tomohisa Hosokawa, Masato Taoka, Koji Tsutsumi, Kanae Ando, Koichi Ishiguro, Masato Hosokawa, Masato Hasegawa, Shin-ichi Hisanaga

**Affiliations:** 1 Laboratory of Molecular Neuroscience, Department of Biological Sciences, Tokyo Metropolitan University, Hachioji, Tokyo 192-0397, Japan; 2 Department of Chemistry, Tokyo Metropolitan University, Hachioji, Tokyo 192-0397, Japan; 3 Juntendo University, Tokyo, 113-0033, Japan; 4 Tokyo Metropolitan Institute of Medical Science, Setagaya, Tokyo 156-8506, Japan.

## Abstract

Tau is hyperphosphorylated in the brains of patients with tauopathies, such as Alzheimer’s disease and frontotemporal dementia and parkinsonism linked to chromosome 17 (FTDP-17). However, neither the mechanism of hyperphosphorylation nor its contribution to pathogenesis is known. We applied Phos-tag SDS-PAGE, a phosphoaffinity electrophoresis, to the analysis of tau phosphorylation *in vitro* by Cdk5, in cultured cells and in mouse brain. Here, we found that Cdk5-p25 phosphorylated tau *in vitro* at Ser404, Ser235, Thr205 and Ser202 in this order. In contrast in cultured cells, Ser404 was preferentially phosphorylated by Cdk5-p35, whereas Thr205 was not phosphorylated. Ser202 and Ser235 were phosphorylated by endogenous kinases. Tau exhibited ~12 phosphorylation isotypes in COS-7 cells with different combinations of phosphorylation at Thr181, Ser202, Thr231, Ser235 and Ser404. These phosphorylation sites were similar to tau phosphorylated in mouse brains. FTDP-17 tau with a mutation in the C-terminal region had different banding patterns, indicating a different phosphorylation pattern. In particular, it was clear that the R406W mutation causes loss of Ser404 phosphorylation. These results demonstrate the usefulness of the Phos-tag technique in the quantitative analysis of site-specific *in vivo* phosphorylation of tau and provide detailed information on *in situ* combinatory phosphorylation of tau.

Tau is a microtubule-associated protein primarily expressed in the axons of neurons. Tau regulates microtubule dynamics and transport of organelles along microtubules in axons^1^,^2^,^3^. These functions of tau are regulated by phosphorylation with a number of protein kinases. Tau is also a major component of neurofibrillary tangles (NFTs) in the brains of patients with tauopathies, including Alzheimer’s disease (AD), frontotemporal dementia and parkinsonism linked to chromosome 17 (FTDP-17)^4^,^5^,^6^. Tau in NFTs is hyperphosphorylated, and therefore, the phosphorylation of tau has been intensively investigated. To date, more than 40 phosphorylation sites have been identified in aggregated tau^7^,^8^,^9^. However, the physiological and pathological roles of the respective phosphorylation sites and how their phosphorylation is regulated are unclear.

Cyclin-dependent kinase 5 (Cdk5) is a proline-directed protein kinase, which phosphorylates Ser or Thr residues followed by Pro; i.e., (S/T)P sequences^10^,^11^. Cdk5 is predominantly activated in neurons by p35 or p39 non-cyclin protein, and regulates a variety of neuronal activities, including neuronal migration, neurite outgrowth, synaptic transmission, plasticity and neuronal survival^12^,^13^,^14^. On the other hand, Cdk5 is abnormally activated when p35 is cleaved to p25 by calpain in neurons undergoing cell death or suffering from stress^15^. Cdk5 is a major tau protein kinase in both physiological and pathological conditions; it is thought that the phosphorylation of tau by Cdk5-p35 is physiological and that by Cdk5-p25 is pathological^16^. Cdk5 phosphorylates mainly Ser202, Thr205, Ser235 and Ser404 *in vitro*^17^,^18^,^19^, although up to 13 sites have been reported to date^7^. While these major Cdk5 sites are phosphorylated in tau from healthy neurons, they are also abnormally phosphorylated in tau from AD brains. Thus, the difference between normal and abnormal phosphorylation is unclear. To resolve this issue, a basic understanding of tau phosphorylation is required, such as which site is phosphorylated to what extent under which conditions. However, it is not clear yet whether the major Cdk5 phosphorylation sites are equally phosphorylated *in vitro* and in cells.

Phosphorylation of proteins has been studied using radioisotope labeling, mass spectroscopy, and anti-phospho-specific antibodies. These methods are powerful to detect phosphorylation but are difficult to use to quantify *in vivo* phosphorylation. To address this issue, Kinoshita *et al.* invented the method Phos-tag sodium dodecyl sulfate polyacrylamide gel electrophoresis (SDS-PAGE)^20^, in which phosphorylated proteins are separated depending on the site and extent of phosphorylation. We have shown that this method is useful in the analysis of *in vivo* phosphorylation of the p35 Cdk5 activator^21^. By applying this method to the analysis of *in vivo* tau phosphorylation, we have recently reported that there is abundant non-phosphorylated tau in mouse and human brains^16^. For the *in vivo* analysis of pathologically phosphorylated tau, however, we needed to determine the physiological phosphorylation states in detail. Here, we used the technique in the analysis of tau phosphorylation by Cdk5 *in vitro* in cultured cells and brain tissues. We identified a number of novel and interesting aspects of the phosphorylation of tau, including FTDP-17 mutant tau, by Cdk5.

## Results

### The ordered phosphorylation of Cdk5-sites in tau *in vitro*

**Figure 1:**
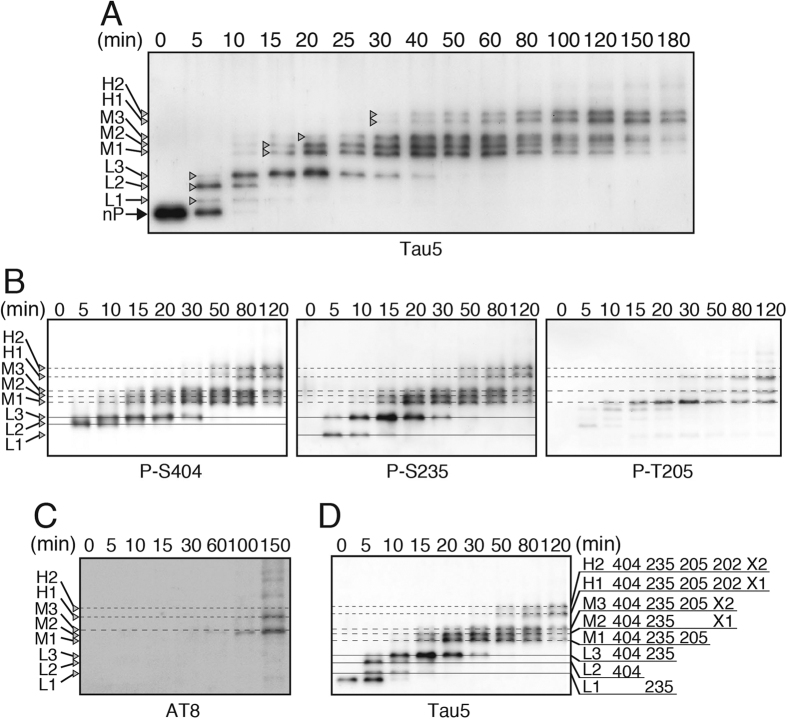
Ordered phosphorylation of tau by Cdk5-p25 *in vitro*. (**A**) Recombinant 1N4R tau was phosphorylated *in vitro* by purified Cdk5-p25 for the indicated times. The phosphorylation profile of tau was analyzed by immunoblotting with Tau5 after Phos-tag SDS-PAGE. Non-phosphorylated tau (nP) with the fastest mobility is indicated by the large arrow at time 0. Tau bands shifted up as the phosphorylation proceeded and they are labeled as L1-3, M1-3 and H1-2 in ascending order. (**B**) Immunoblotting of tau phosphorylated by Cdk5-p25 for the indicated times with anti-phospho-Ser404 (P-S404), anti-phospho-Ser235 (P-S235), and anti-phospho-Thr205 (P-T205). L1-3 are indicated by thin-solid lines, M1-3 are dotted lines and H1-2 are dotted-and-dashed lines. (**C**) Immunoblotting of tau with AT8, which recognizes tau doubly phosphorylated at Ser202 and Thr205. (**D**) Assignment of phosphorylation sites in tau L1-3, M1-3 and H1-2, which were separated using Phos-tag-SDS-PAGE. Unidentified sites, which are supposed to be in M2/H1 and M3/H2, are designated as X1 and X2, respectively.

Cdk5 phosphorylates tau *in vitro* primarily at four sites, Ser202, Thr205, Ser235 and Ser404^17^,^18^,^19^. However, it is not known whether they are phosphorylated randomly or in an orderly fashion. Further, it is not clear whether Cdk5 targets all of those sites on a single tau molecule processively. To address these questions, we used Phos-tag SDS-PAGE. Recombinant tau was incubated with Cdk5-p25 in the presence of ATP, and phosphorylation was assessed by upward shift on an immunoblot generated using Phos-tag SDS-PAGE and the phosphorylation-independent tau antibody Tau5. Tau shifted up gradually through several discrete bands with incubation time (Fig. 1A). Fully phosphorylated tau was not observed together with non- or low-phosphorylation states of tau at any incubation time, indicating that tau molecules are synchronously phosphorylated in a particular order in the *in vitro* reaction conditions.

To establish the order of phosphorylation of the sites, tau at the different incubation times was blotted with anti-phospho-specific antibodies against respective Cdk5 sites (Fig. 1B). The non-phosphorylated recombinant tau band (Fig. 1A, nP in the time 0 lane) decreased and three bands labeled as L1-3 appeared at 5 min, with L2 being the strongest in the Tau5 blot. L2 was reactive against anti-P-S404 and L1 was reactive against anti-P-S235, indicating that Ser404 is phosphorylated first and Ser235 second, although Ser235 can be phosphorylated first in a small percent of tau molecules. L3 was stronger at 10–20 min and reacted with both anti-P-S404 and P-S235, suggesting that L3 was doubly phosphorylated tau at Ser235 and Ser404. Next, two bands, M1 and M2, appeared almost identically at 15 min, while band M3 was slightly delayed at 20 min on the Tau5 blot. M1 was reactive with anti-P-T205, indicating that M1 is additionally phosphorylated at Thr205. To identify the bands containing phospho-Ser202, we used anti-P-S202 (Abcam), which specifically reacted with the phospho-Ser202 of tau wild type (WT), but not the Ala mutant, in COS-7 cells (Supplemental Fig. 1, upper panel). The reaction was not observed for *in vitro* Cdk5-phosphorylated tau because its reaction was lost by phosphorylation at Thr205 (Supplemental Fig. 1, lower panel). To understand the phosphorylation at Ser202, we used AT8, which recognizes phosphorylated tau at both Ser202 and Thr205. AT8 reacted with H1 and H2 and a band at M3 after a 150 min incubation (Fig. 1C). There are several faint bands above H2 in the blot of AT8. There would be tau phosphorylated at minor phosphorylation sites that occurred after the major sites were phosphorylated. The band at M3 was also reactive with anti-P-T205 but appeared to be different from the major M3 band detected with anti-P-S235 and P-S404 at 20 min. Because we could not identify additional phosphorylation sites in M2 and M3, they were tentatively labeled as X1 and X2, respectively (Fig. 1D). Thus, H1-2 appeared last, representing additional phosphorylation at Ser202 to M1 and M2. The phosphorylation sites in each band are indicated in Fig. 1D. These results indicate that Cdk5-p25 phosphorylates tau in the order Ser404, Ser235, Thr205 and Ser202.

### Site-specific phosphorylation of tau by endogenous kinase(s) and/or Cdk5-p35 in COS-7 cells

**Figure 2:**
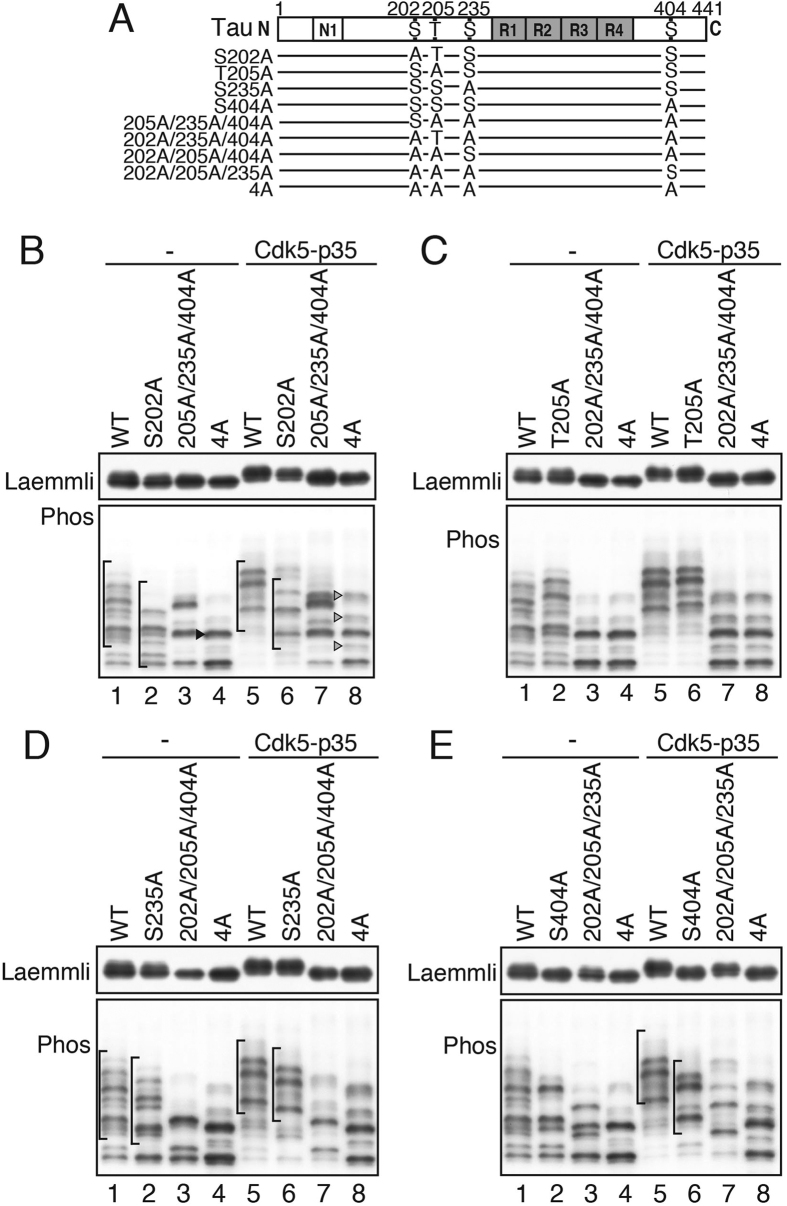
*In situ* phosphorylation of tau WT and its alanine mutants by Cdk5 in COS-7 cells. (**A**) Major Cdk5 phosphorylation sites in tau and their alanine mutants. Amino acids are numbered according to the longest human tau isoform 441. (**B**)~(**E**) Tau and its alanine mutants were individually expressed in COS-7 cells in the absence (−) or presence of Cdk5-p35. Their phosphorylation-dependent shifts were examined by immunoblotting with Tau5 after Laemmli’s (upper) or Phos-tag SDS-PAGE (lower). (**B**) S202A and 205A/235A/404A, (**C**) T205A and 202A/235A/404A, (**D**) S235A and 202A/205A/404A, and (**E**) S404A, and 202A/205A/235A. Arrowhead in lane 4 of (**B**) indicates a band of tau 4A with a single phosphorylation at a non-Cdk5 site. Arrowheads in lane 8 of (**B**) indicate bands of tau 4A that were phosphorylated by Cdk5-p35 at sites other than the four major Cdk5-sites. Brackets in (**B,D,E**) indicate a group of bands that shifted down their mobility as a whole by an Ala mutation of Ser202 (**B**), Ser235 (**D**) and Ser404 (**E**). Immunoblottings of tau after Laemmli’s SDS-PAGE were performed under the same experimental conditions as an example of the uncropped image, which is provided in Supplemental Fig. 3a.

Next, we examined how these Cdk5 sites are phosphorylated in cellular conditions using mutants in which one or more of the sites are substituted with an alanine. To characterize site-specific phosphorylation of tau in cells, we prepared two sets of mutants: the 1A mutants, for which each member had one of the four sites mutated to an alanine, and the 3A mutants, for which different sets of three sites were mutated to alanine (Fig. 2A). Tau and the 1A and 3A mutants were individually expressed in COS-7 cells in the absence or presence of Cdk5-p35, and their phosphorylation-dependent upward shifts were examined by immunoblotting with Tau5 after Phos-tag SDS-PAGE (Fig. 2B–E, lower panels). In this experiments, we used a 1N4R isoform of tau and confirmed the absence of cleavage of tau, which might affect the banding pattern in Phos-tag SDS-PAGE, by Laemmli’s SDS-PAGE (Fig. 2B–D, upper panels).

In the 4A mutant (lanes 4 and 8 in Fig. 2B–E), tau 4A separated into two major bands (arrowheads in lane 4 of Fig. 2B) when expressed alone in COS-7 cells. The lower band corresponded to non-phosphorylated tau, which co-migrated with recombinant 1N4R tau expressed in *E. coli* (data not shown), and the upper band might have a single phosphorylation at a particular non-Cdk5 site. About half of tau 4A was phosphorylated at the site in COS-7 cells. The minor bands slightly increased in intensity with co-expression of Cdk5-p35 (arrowheads in lane 8 of Fig. 2B), suggesting that they are also phosphorylated partly by Cdk5-p35 in COS-7 cells and that Cdk5-p35 phosphorylates a few other sites to some degree.

**Figure 3:**
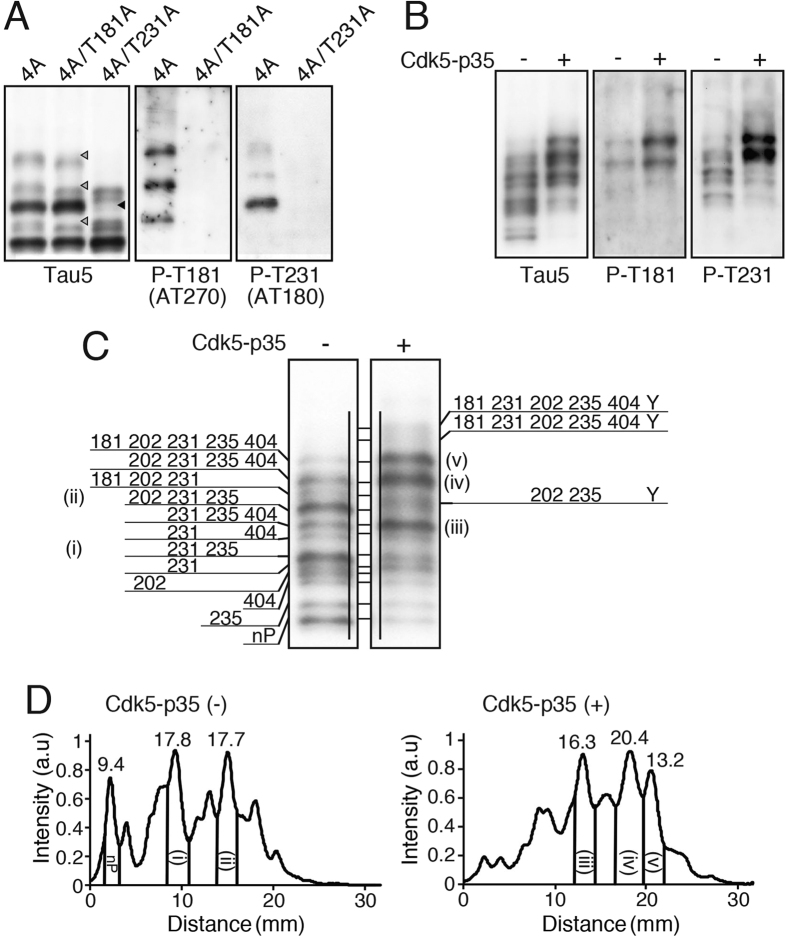
Phosphorylation sites assigned in each band of tau expressed in COS-7 cells. (**A**) Identification of Thr231 as a major and Thr181 as a minor phosphorylation site in tau 4A in COS-7 cells. Tau 4A, 4A/T181A (4A with Thr181-to-Ala mutation) or 4A/T231A (4A with Thr231-to-Ala mutation) was expressed in COS-7 cells and immunoblotted with Tau5, AT180 (P-T231) and AT270 (P-T181) after Phos-tag SDS-PAGE. Arrowheads indicated the band(s) disappearance with 4A/T181A or 4A/T231A mutant. (**B**) Immunoblotting of tau WT expressed in COS-7 cells in the absence (−) or presence (+) of Cdk5-p35 with anti-P-T181 or anti-P-T231. (**C**) Phosphorylation site assignment in respective bands of tau, which was expressed in COS-7 cells in the absence (−) or presence (+) of Cdk5-p35 and separated by Phos-tag SDS-PAGE. nP is non-phosphorylated tau. Unidentified phosphorylation site(s), which appeared as co-expressed with Cdk5-p35, are indicated collectively by Y. (**D**) Densitometric scans of the banding patterns of tau in the absence (left) and presence (right) of Cdk5-p35. The percent ratios of several major bands to the total tau are indicated above the respective peaks.

Tau WT separated into ~12 bands in COS-7 cells (lane 1 of Fig. 2B and see also Fig. 3C). The slowly migrating tau bands indicated by the bracket shifted downward, keeping a similar arrangement of the bands, when Ser202 was mutated to alanine (Fig. 2B, lanes 1 and 2). Adding Ser202 to tau 4A shifted the two major bands of 4A up (Fig. 2B, lanes 3 and 4). These results indicate clearly that Ser202 was phosphorylated by endogenous kinase(s) in COS-7 cells, and these upper bands are phosphorylated at Ser202. In addition, exogenous Cdk5-p35 reduced the extent of the non-phosphorylated band of 205A/235A/404A mutant, resulting in an increase in the signal of the upper bands by phosphorylation (Fig. 2B, compare lanes 3 and 7), indicating that Ser202 can also be phosphorylated by Cdk5-p35 in cells.

The Thr205-to-Ala mutation did not change the banding pattern in COS-7 cells or in the presence of Cdk5-p35, indicating that Thr205 is hardly phosphorylated by endogenous kinase(s) or by Cdk5-p35 in cultured cells (Fig. 2C). The Ala mutation at Ser235 shifted down in the absence of Cdk5-p35 (compare lanes 1 and 2 or lanes 3 and 4 in Fig. 2D) and Cdk5-p35 expression decreased the non-phosphorylated band (Fig. 2D, lanes 3 and 7). In addition to Ser202, Ser235 was phosphorylated by endogenous kinase(s) (Fig. 2D, bracket in lanes 1 and 2) and also by Cdk5-p35 in COS-7 cells. In contrast, the S404A mutation altered the banding pattern of tau in the presence of Cdk5-p35 more than the absence (Fig. 2E, lanes 1 and 2; lanes 5 and 6). The positions of 202A/205A/235A mutant shifted completely when Cdk5-p35 was expressed in the cells (Fig. 2E, lanes 3 and 7). Thus, Ser404 is preferentially phosphorylated by Cdk5-p35 (Fig. 2E, lanes 7 and 8), although a portion can be phosphorylated in COS-7 cells in the absence of Cdk5-p35 (Fig. 2E, lane 3). These results indicate that Ser202 and Ser235 are phosphorylated largely by endogenous kinase in COS-7 cells, and Ser404 is phosphorylated mainly by Cdk5, but Thr205 is not targeted by endogenous kinase(s) or Cdk5.

These experiments enabled us to assign Ser202, Ser235 and Ser404 to respective tau bands in COS-7 cells. To assign phosphorylation sites completely, however, we needed to identify the one unknown major phosphorylation site found in tau 4A (Fig. 2B, lane 4). Upon searching the literature^17^,^22^,^23^, Thr181 and Thr231 were identified as candidates. We made 5A constructs by adding a mutation at Thr181 or Thr231 to tau 4A, and their phosphorylation was examined by Phos-tag SDS-PAGE (Fig. 3A). The upper major band of tau 4A was lost by the Ala mutation at Thr231 (black arrowhead in lane 3 of Fig. 3A) and several minor bands disappeared by adding an Ala mutation at Thr181 (white arrowheads in lane 2 of Fig. 3A). These results were confirmed by immunoblotting with anti-P-T181 (Fig. 3A, middle) and anti-P-T231 (Fig. 3A, right), respectively. Using these two phosphorylation site-specific antibodies, we examined which tau bands in COS-7 cells contained phospho-231 and phospho-181. Anti-P-T231 reacted with more than half of the tau bands with slower migration and co-expression of Cdk5-p35 shifted up the P-T231-containing bands (Fig. 3B, P-T231). Anti-P-T181 reacted with upper two bands and their position did not change with co-expression of Cdk5-p35 (Fig. 3B, P-T181).

From these data, we assigned most phosphorylation sites to each tau band detected in COS-7 cells. There are approximately 12 bands, including minor ones, for tau with different phosphorylation combinations in the absence of Cdk5-p35 (Fig. 3C, left). The phosphorylation sites detected were Thr181, Ser202, Thr231, Ser235 and Ser404, which are all proline-directed sites. We identified tau with a single phosphorylation to a maximum of five phosphorylations. Co-expression of Cdk5-p35 increased the intensity of the bands containing Ser404, or shifted the bands upward by additional phosphorylation at Ser404 (Fig. 3C, right). An unidentified minor Cdk5 phosphorylation site is designated as Y (Fig. 3C, right). The densitometric scanning profiles are shown below. We quantified several major bands: 9.4% for unphosphorylated tau, 17.8% for doubly phosphorylated tau at Thr231 and Ser235, 17.7% for tau with triple phosphorylation at Ser202, Thr231 and Ser235 in the absence of Cdk5-p35, 16.3% for tau with phosphorylation at Thr231, Ser235 and Ser404, 20.4% for tau with four sites at Ser202, Thr231, Ser235 and Ser404, and 13.2% for tau with five sites at Thr181, Ser202, Thr231, Ser235 and Ser404 (Fig. 3D). We also calculated the ratio of phosphorylation at Thr231 because all eight upper bands were phosphorylated. Thr231 was phosphorylated in approximately 73.3% of tau expressed in COS-7 cells. Thr231 is phosphorylated by GSK3β^24^,^25^, suggesting that GSK3β is highly active in COS-7 cells. Considering that GSK3β is also a proline-directed kinase, some of Cdk5 sites could be phosphorylated by GSK3β.

### Tau phosphorylated by Cdk5-p35 and Cdk5-p25

**Figure 4:**
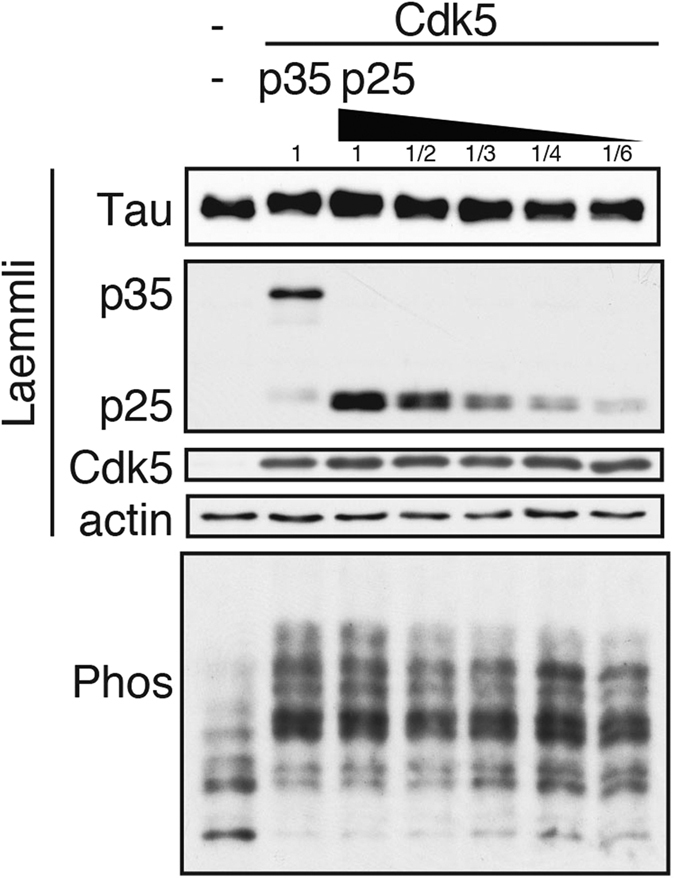
Tau phosphorylation by Cdk5-p35 or Cdk5-p25 in COS-7 cells. COS-7 cells were co-transfected with plasmids encoding tau WT, Cdk5, and p35 or various concentrations of p25. The phosphorylation of tau was detected by immunoblotting with Tau5 after Laemmli’s (top) or Phos-tag SDS-PAGE (bottom). Cdk5 and p35 or p25 were detected by immunoblotting with anti-Cdk5 and anti-p35/p25 antibodies. Actin was the loading control. Immunoblottings of tau, Cdk5, p35 and actin after Laemmli’s SDS-PAGE were performed under the same experimental conditions as examples of the uncropped images, which are provided in Supplemental Figure 3a,b.

Cdk5 is overactivated by cleavage of p35 to p25 with calpain in neurons^26^,^27^,^28^. We compared tau phosphorylation by Cdk5-p35 and Cdk5-p25 in cultured cells using Phos-tag SDS-PAGE. The level of Cdk5 kinase activity is determined by the available amounts of the activators. Because p35 is degraded more rapidly than p25, the amount of p35 was considerably less than that of p25 when the same amounts of the plasmids were used for transfection^29^. Therefore, the phosphorylation patterns of tau were compared by changing the expression levels of p25 (Fig. 4). By increasing the expression of p25, the phosphorylation of tau was increased, as shown by the higher upward shift. However, the upward shift of tau did not exceed those phosphorylated by Cdk5-p35. These results indicate that Cdk5-p25 phosphorylates tau to a similar extent to Cdk5-p35, at least in COS-7 cells.

### Phosphorylation of P301L and R406W mutant tau by Cdk5-p35 in COS-7 cells

**Figure 5:**
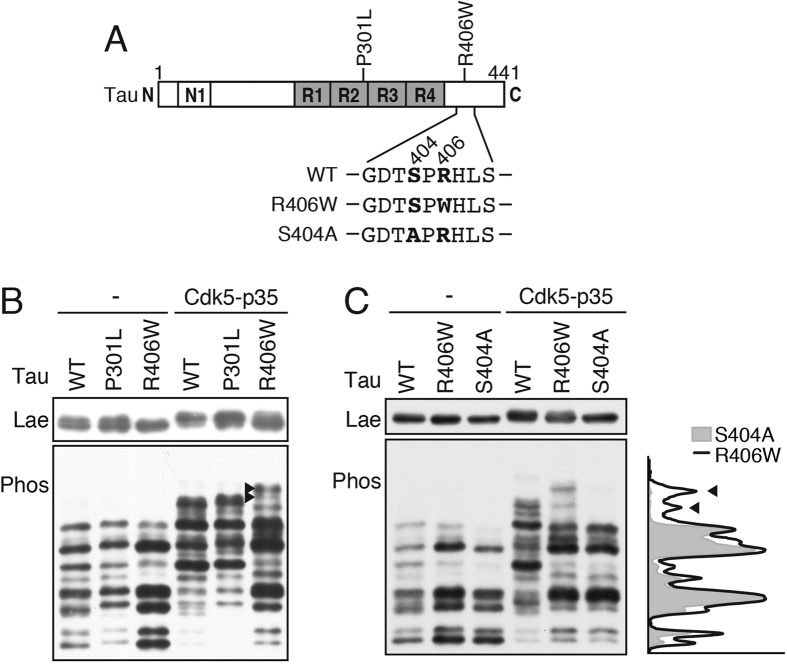
Phosphorylation of tau P301L and R406W mutants by Cdk5-p35. **(A**) Positions of P301L and R406W mutations in the tau molecule and amino acid sequences 401–410 of tau WT and R406W and S404A. The Ser404 phosphorylation site and Arg406 mutation site are shown with bold letters. (**B**) Phos-tag SDS-PAGE analysis of tau WT, P301L and R406W expressed in COS-7 cells with or without co-expression of Cdk5-p35. (**C**) Comparison of banding pattern of R406W and S404A expressed in COS-7 cells with or without Cdk5-p35. Tau was detected by immunoblotting with Tau5. The upper is Laemmli’s SDS-PAGE and the lower is Phos-tag SDS-PAGE. Densitometric scans of R406W (black line) and S404A (light gray) in the presence of Cdk5-p35 are shown in the right side of the blot. Arrows indicate the bands specifically detected with R406W. Immunoblottings of tau after Laemmli’s SDS-PAGE were performed under the same experimental conditions as an example of the uncropped image, which is provided in Supplemental Fig. 3a.

FTDP-17 tau is causative for FTDP-17 disease and is hyperphosphorylated in diseased brains^6^,^30^,^31^, but the mechanism(s) of hyperphosphorylation is unknown. We investigated the phosphorylation states of FTDP-17 tau mutants in the presence and absence of Cdk5-p35 in COS-7 cells. We initially characterized tau P301L and R406W (Fig. 5A) not only because they are representative mutants with different symptoms in early and late onset disease^6^,^30^,^32^,^33^ but also because we have previously analyzed their Cdk5 phosphorylation^18^,^19^. P301L had an identical phosphorylation pattern to that of tau WT in the presence or absence of Cdk5-p35 (Fig. 5B). In contrast, although R406W had a banding pattern similar to WT in the absence of Cdk5-p35, in the presence of Cdk5-p35 the banding pattern was altered considerably, such that the major phosphorylation site appeared to be lost. Nonetheless, there was a highly phosphorylated band in R406W (Fig. 5B, arrowhead), which migrated more slowly than any bands of tau WT and P301L in Phos-tag SDS-PAGE.

Ser404 was the preferential Cdk5 phosphorylation site (Fig. 2). Cdk5 prefers (S/T)P sequences with a basic amino acid in the C-terminal + 3 site^34^,^35^. Exchange of the basic amino acid Arg to Trp at the + 2 site by the R406W mutation may result in decreased phosphorylation at Ser404. Therefore, we asked if the phosphorylation pattern of R406W is or is not identical to that of S404A. All of tau WT, R406W and S404A showed similar, but not identical, banding patterns (Fig. 5C) when phosphorylated by endogenous kinases in COS-7 cells. On the other hand, co-expression with Cdk5-p35 substantially changed the patterns of R406W and S404A in comparison to tau WT, whereas the phosphorylation patterns of R406W and S404A were very similar (Fig. 5C), indicating that the R406W mutation abolishes Ser404 phosphorylation by Cdk5. Nonetheless, the banding patterns were not exactly identical between R406W and S404A. Their densitometric scanning results are shown in the right side of Fig. 5C and reveal several highly phosphorylated species specific to R406W. The results indicate that R406W mutation alters the phosphorylation of tau by not only losing one major site at Ser404 but also gaining at least one additional site that is not found in tau WT.

### Phosphorylation of other FTDP-17 tau mutants with mutation in the N-terminal, MTB and C-terminal regions

**Figure 6:**
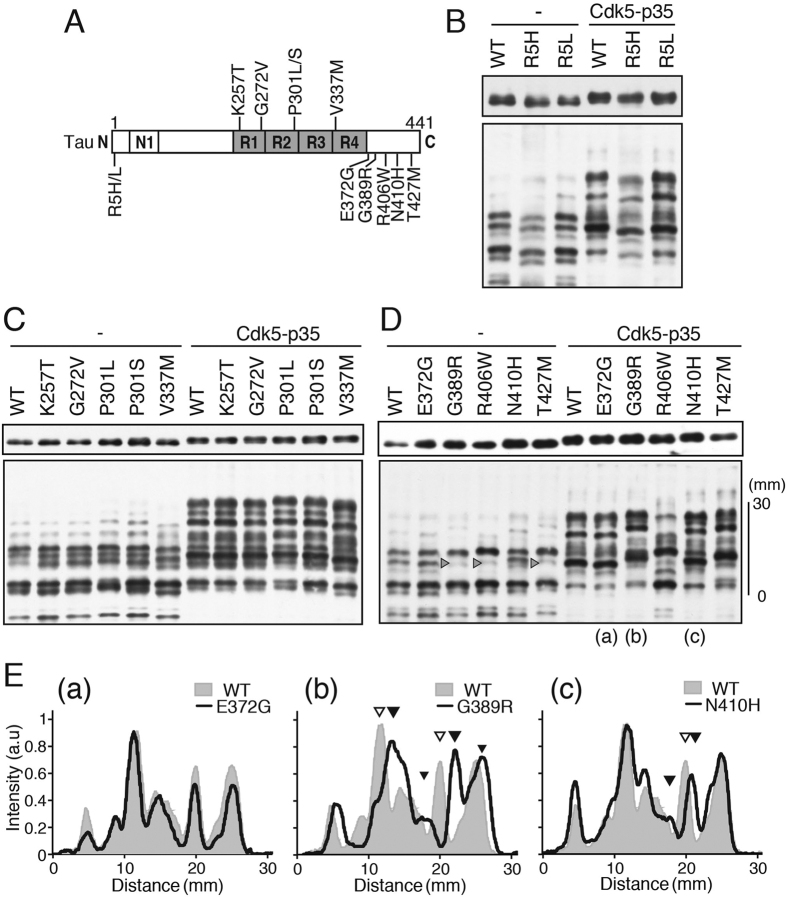
Phosphorylation of tau with the FTDP-17 mutation in the N-terminal, MTB and C-terminal regions. **(A**) Positions of FTDP-17 mutations in the tau molecule examined here. (**B–D**) COS-7 cells were transfected with the indicated plasmid encoding tau WT or one of the FTDP-17 mutants in the absence (−) or presence (+) of Cdk5-p35. The phosphorylation of tau was examined by immunoblotting with anti-human tau after Laemmli’s (upper) or Phos-tag SDS-PAGE (lower). (**B**) The N-terminal mutants R5H and R5L. (**C**) Tau mutants in the MTB region, K257T, G272V, P301L, P301S, and V337M. (**D**) The C-terminal mutants E372G, G389R, R406W, N410H and T427M. Arrowheads in the lanes of G389R, R406W and T427M indicate a band whose intensity was reduced in these mutants in the absence of Cdk5-p35. (**E**) Densitometric comparison of the banding patterns between tau WT and E372G (a), G389R (b) or N410H (c) expressed in COS-7 cells in the presence of Cdk5-p35, indicated by (a)~(c) in (**D**). Tau WT is shown in light gray, and E372G, G389R and N410H are shown by a black line. Arrowheads indicate the major peaks shifted in mutants. Immunoblottings of tau after Laemmli’s SDS-PAGE were performed under the same experimental conditions as an example of the uncropped image, which is provided in Supplemental Fig. 3a.

FTDP-17 mutations are distributed throughout tau, although the majority is found in the microtubule-binding (MTB) region^6^,^36^. We examined here the phosphorylation of tau with mutations in the N-terminus using R5H or R5L, in the MTB region with K257T, G272V, P301L/S and V337M as representatives, and in the C-terminal region using E372G, G389R, R406W, N410H and T427M (Fig. 6A). Each mutant was co-transfected with or without Cdk5-p35 in COS-7 cells and the phosphorylation patterns were compared to that of tau WT by Phos-tag SDS-PAGE. The N-terminal and MTB mutations did not affect the banding pattern either in the absence or presence of Cdk5-p35 (Fig. 6B,C). In contrast, several C-terminal mutants displayed different banding patterns from tau WT (Fig. 6D). E372G, with a mutation only 4 amino acids downstream of the C-terminal end of MTB, had an identical pattern to tau WT (Fig. 6E, a). R406W was described above. The G389R and T427M mutants had the same banding pattern, which was different from those of tau WT or R406W (Fig. 6D). In the absence of Cdk5-p35, the second band from the top was greatly decreased, as well as R406W (Fig. 6D, arrowheads), but the banding pattern was quite different from that of R406W in the presence of Cdk5-p35. The banding pattern of G389R was compared to that of tau WT and revealed that two major peaks shifted upward slightly (Fig. 6E, b, black arrowhead). N410H was also slightly different from tau WT (Fig. 6D). The band shift was observed at the peak indicated by arrowheads in Fig. 6E (c), which correspond to the upper one of two peaks shifted in G389R (arrowhead in Fig. 6E, b). The data suggest that G389R, N410H and T427M mutations may slightly change the phosphorylation state of tau.

Because the changes in the banding pattern were more apparent during co-expression with Cdk5-p35, we thought it possible that phosphorylation at Cdk5 sites was affected by these C-terminal mutations. To identify which phosphorylation site is affected by these C-terminal mutations, we examined phosphorylation at the major Cdk5 sites in COS-7 cells by two-dimensional phospho-peptide map analysis (Supplemental Fig. 2b) and immunoblotting with anti-phospho-specific antibodies (Supplemental Fig. 2a). Using these methods, we did not detect clear differences in phosphorylation between tau WT and the C-terminal mutants. These mutations may affect minor Cdk5 phosphorylation sites or non-Cdk5 sites.

### Tau in mouse brain has a phosphorylation profile similar to COS-7 cells

**Figure 7:**
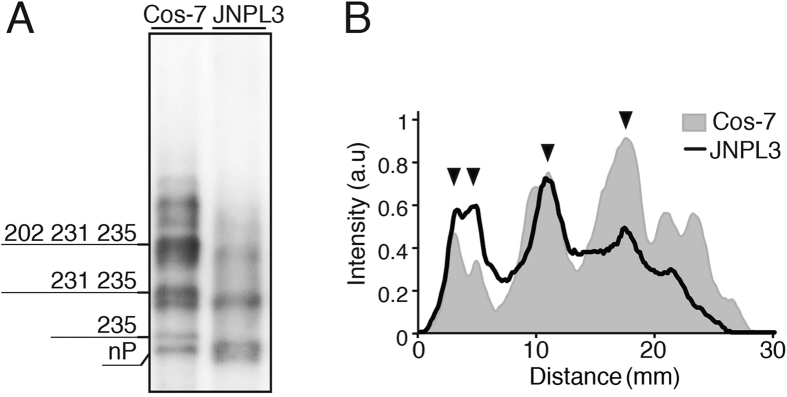
Comparison of phosphorylation profiles of tau expressed in mouse brain and COS-7 cells by Phos-tag SDS-PAGE. (**A**) Immunoblotting of human tau WT (0N4R) expressed in COS-7 cells (left lane) and tau P301L (0N4R) in JNPL3 transgenic mouse (right lane) with Tau5 after Phos-tag SDS-PAGE. Phosphorylation sites of major tau bands expressed in COS-7 cells are indicated. (**B**) Densitometric comparison of the banding patterns between tau in COS-7 cells and mouse brain.

COS-7 cells are a non-neuronal cell line. It is important to determine whether tau in the brain has a phosphorylation profile similar to tau in cultured cells. Because mouse tau is nine amino acids shorter than human tau, and multiple isoforms of tau are expressed in the brain, we decided to analyze the phosphorylation of human tau expressed in mouse brain using the JLPL3 P301L transgenic mouse. It was shown above that P301L tau has the identical phosphorylation profile to tau WT in COS-7 cells (Fig. 5B). Because the P301L transgene encodes 0N4R tau (an isoform of tau with no N-terminal insertion and with four microtubule repeats), we first confirmed that 0N4R tau is phosphorylated identically to 1N4R tau in COS-7 cells (data not shown). We used soluble tau prepared from mouse brains at 24 weeks^16^. The phosphorylation of human tau was compared between COS-7 cells and mouse brain tissue by Phos-tag SDS-PAGE (Fig. 7). Densitometric scanning of the banding patterns are shown in Fig. 7B. Positions of several major bands are similar between them, although the intensity was different. Tau, indicated by arrowheads in Fig. 7B, was phosphorylated at S202/T231/S235 or T231/S235, or unphosphorylated. Thus, the results obtained from COS-7 cells can be adapted to phosphorylation of tau in the brain.

## Discussion

In this report, we characterized the phosphorylation of tau by Cdk5 *in vitro*, in cultured cells and in brain tissue using the Phos-tag SDS-PAGE technique. Cdk5 phosphorylated tau at Ser404, Ser235, Thr205 and Ser202 in this order *in vitro*. Among these major *in vitro* phosphorylation sites, Ser202 and Ser235 were phosphorylated by endogenous kinase(s) in COS-7 cells. Tau occurs as ~12 different phosphorylation isotypes in COS-7 cells, with a maximum of five phosphorylation sites, all of which are proline-directed sites. Ser404 was phosphorylated preferentially by Cdk5-p35, but Thr205 was rarely phosphorylated. Mouse brain tau showed some similarity in phosphoisotypes, but not the extent of phosphorylation, to those of tau expressed in cultured cells. The C-terminal mutations of FTDP-17 tau affected the banding pattern of tau in Phos-tag SDS-PAGE. In particular, it was clearly shown that the R406W mutation eliminated phosphorylation at Ser404. These results increase greatly our understanding of physiological and pathological phosphorylation of tau.

**Figure 8:**
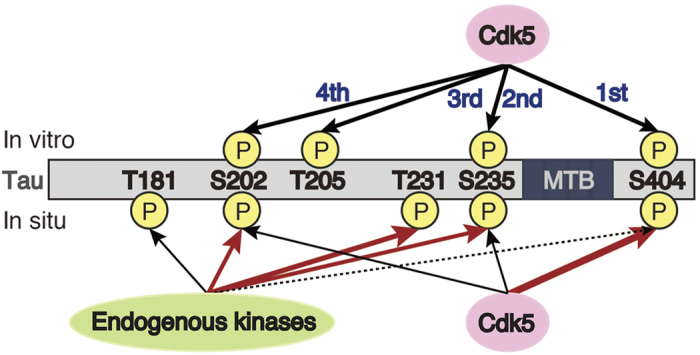
*In vitro* and *in situ* phosphorylation of tau by Cdk5. Cdk5-p25 phosphorylates tau mainly at Ser404, Ser235, Thr205 and Ser202 in this order *in vitro*. Major phosphorylation sites of tau expressed in COS-7 cells are Ser202, Thr231, Ser235, and Ser404 with one minor site at Thr181. Among *in vitro* Cdk5 sites, Ser202 and Ser235 are phosphorylated by endogenous kinase(s). While Ser404 was preferentially phosphorylated by Cdk5-p35, Thr205 was rarely phosphorylated in COS-7 cells. Thr231 is phosphorylated in more than half of tau molecules in cells. Thr181 is also phosphorylated in relatively highly phosphorylated species of tau.

Previous studies have identified 11~13 phosphorylation sites for Cdk5 in tau^7^,^8^, but this does not necessarily mean that all sites are equally phosphorylated or in a single tau molecule. To understand the phosphorylation site-specific function precisely, it is important to know not only which kinase phosphorylates which site but also which site is phosphorylated to what extent. We have confirmed Ser202, Thr205, Ser235 and Ser404 as major *in vitro* Cdk5 sites^17^,^18^,^37^. Here, using Phos-tag SDS-PAGE and phospho-specific antibodies, we have shown that tau was sequentially phosphorylated *in vitro* by Cdk5-p35 in the order of Ser404, Ser235, Thr205 and Ser202 (Fig. 8). Although the order is from the C-terminus to the N-terminus, the phosphorylation does not proceed sequentially on a single tau molecule Instead, Ser404 is first targeted in individual tau molecules and then Ser235 is attacked. Considering that tau is an extended and essentially unfolded molecule, the order of phosphorylation may be determined simply by the amino acid sequence around the phosphorylation sites^37^.

By using Phos-tag SDS-PAGE and site-specific Ala mutants, we showed that *in vitro*, Cdk5 phosphorylation sites are not necessarily phosphorylated by Cdk5-p35. Ser202 and Ser235 were phosphorylated by endogenous kinase(s) in the absence of Cdk5 activity (Fig. 8). COS-7 cells express Cdk5 protein but do not display its kinase activity because its activators are not expressed. In this study, we co-transfected Cdk5 with p35 to obtain sufficient activity of Cdk5-p35. These two sites are phosphorylated by other proline-directed protein kinases (PDPKs), such as glycogen synthase kinase 3β (GSK3β) or MAP kinases, ERK, JNK and p38. Although we have not identified the responsible protein kinase in COS-7 cells in this study, these sites might be targeted by other PDPKs^7^,^8^. In contrast, Thr205 was hardly phosphorylated in COS-7 cells whether or not Cdk5-p35 was co-expressed. Although this site is also reported to be phosphorylated by any one of the PDPKs, its phosphorylation property contrasts to those of Ser202 and Ser235. We have shown previously that Ser202 and Thr205 are incompatible *in vitro* phosphorylation sites^19^. However, different from the *in vitro* phosphorylation, where Thr205 was phosphorylated in S202A mutant tau by Cdk5, it was not phosphorylated in the 3A mutant, including the S202A mutation, in cells (Fig. 2C). One of differences between *in vitro* and in cells is the association with microtubules, which affects phosphorylation at Ser202 and Thr205^38^. Thr205 is shown to be hyperphosphorylated in AD brains because its phosphorylation generates an AT8 epitope together with phosphorylation at Ser202^39^. AT8 is one of most frequently used anti-phospho-tau antibodies for the diagnosis of AD pathology. However, it is not clear how the AT8 reactivity is generated in AD brains. Phosphorylation at Thr205 may discriminate AD pathology from physiological tau phosphorylation. It may be important to identify cellular conditions under which Thr205 is phosphorylated.

Ser404, the site phosphorylated first by Cdk5-p35 *in vitro*, was preferentially phosphorylated by Cdk5 in cells, although the site is also reported to be phosphorylated by other PDPKs^7^,^8^. Thus, cellular phosphorylation properties are not equivalent *in vitro* among Cdk5 sites in tau. If Ser404 is a Cdk5 specific site even in brains, its phosphorylation would be an indication of the Cdk5 activity targeting to tau. The previous results indicate that Ser404 is one of major phosphorylation sites in the brain of young and adult rats^22^,^40^. We have recently reported that more than half of tau is phosphorylated at the site in normal aged human brains^16^. These data suggest that Cdk5 is a major physiological tau kinase^17^. On the other hand, Ser404 phosphorylation constitutes a pathological epitope of PHF-1 with Ser396 phosphorylation^41^. If Ser404 is phosphorylated physiologically, the PHF-1 reactivity would be generated by additional phosphorylation at Ser396 with GSK3β^42^. This idea is consistent with sequential phosphorylation of tau by GSK3β at the primed sites by Cdk5 in production of the AD pathological phosphorylation epitopes^17^,^43^,^44^.

Interestingly, Ser404 phosphorylation was almost completely abolished by the R406W mutation. There have been many studies of the phosphorylation of the R406W mutant. Most of them report unique phosphorylation of R406W tau, but the results are inconsistent; reduced phosphorylation at most sites^45^; reduced phosphorylation at specific sites^46^,^47^ or greater overall phosphorylation^48^. Our data showed clearly that a consequence of the R406W mutation resulted in the elimination of Ser404 from the Cdk5 site. This was indicated by the almost identical phosphorylation pattern of R406W to that of S404A when Cdk5 was active. Cdk5 prefers (S/T)P sites with a basic residue usually two residues downstream of the (S/T)P sequence^34^,^35^. Mutation of Arg406 to tryptophan, while it is only one residue downstream, would disrupt the Cdk5-preferred consensus SPR sequence (Fig. 5A). Nevertheless, the R406W mutant showed several highly phosphorylated bands above those of the S404A mutant or even tau WT. The R406W mutation may have an additional effect on the structure of tau, which would increase phosphorylation at unknown sites. Identification would lead to the unveiling of the R406W pathology. In any cases, previous inconsistencies could be due to the experimental conditions, whether Cdk5 was used as a kinase source or not, or the phosphorylation sites examined.

FTDP-17 tau mutants are hyperphosphorylated in the brains of patients^31^,^49^, but such hyperphosphorylation has not been seen distinctly when they are phosphorylated *in vitro* or in cultured cells^18^,^45^,^50^,^51^. However, only a limited number of FTDP-17 mutants have been examined previously. In particular, at least to our knowledge, the phosphorylation patterns of the N-terminal mutants and C-terminal mutants, except for R406W, have not been examined. We confirmed that the FTDP-17 tau with a mutation in the MTB region had an identical phosphorylation pattern to tau WT whether or not they were co-expressed with Cdk5-p35 (Fig. 6C). The N-terminal mutants of R5H and R5L also had an identical banding pattern to tau WT (Fig. 6B). In contrast, three C-terminal mutants, G389R, N410 and T427M, had a slightly different banding pattern to tau WT. Because the difference was obvious when they were phosphorylated by Cdk5-p35 (Fig. 6D), we thought the mutation might affect Cdk5 phosphorylation. However, we did not detect a difference in phosphorylation by immunoblotting with anti-phospho antibodies or 2D-phosphopeptide mapping. Considering the properties of Phos-tag SDS-PAGE, it could be that minor Cdk5 phosphorylation sites may be affected, as was observed with R406W tau. If this is the case, the question arises as to how these mutations affect phosphorylation pattern slightly. One possibility is a conformational change of tau; a partial conformation is suggested for tau^52^,^53^,^54^. In addition, it may be worth noting that the C-terminal truncation at Glu391 or Asp421 may associate with AD^55^,^56^. Truncated tau is reported to be prone to aggregate or to be secreted than tau WT. The C-terminal mutation may induce conformational changes similar to those induced by the C-terminal truncation.

Tau occurs as ~12 phosphorylation isotypes in COS-7 cells. Some of them showed the similar migration to tau of brains, suggesting that tau is phosphorylated at the same sites in the brain as in cultured cells; those sites are Ser202, Thr231 and Ser235. It was interesting that there are many different phosphorylation isotypes in cultured cells and brains. In the case of cultured cells, the cell cycle may produce heterogeneities of phosphorylation. It was shown that tau is highly phosphorylated in the M phase^57^, when Cdk1/cyclin B with a similar substrate specificity to Cdk5 is activated. In neurons without a cell cycle, types of neurons, excitation and intracellular distribution may affect the phosphorylation of tau. It would be intriguing to study tau with different phosphorylation sites in neurons.

In summary, we analyzed tau phosphorylation using the Phos-tag technique and identified site-specific and combinatorial phosphorylation of tau. Tau is heterogeneously phosphorylated in cells. (Phos-tag is phospho-affinity electrophoresis that does not recognize other protein modification such as acetylation) Nevertheless, we have assigned phosphorylation sites in different phosphorylated states in cultured cells. These results demonstrate that the Phos-tag would be a powerful method to characterize *in vivo* physiological and pathological phosphorylation of tau.

## Methods

### Antibodies and Plasmids used

Anti-tau Tau5 and AT270 (P-T181) were purchased from ThermoFisher Scientific (Fremont, CA). Anti-human tau Tau-12 was described previously^58^. Anti-phospho-Ser202, phospho-Ser231, phospho-Ser235 and phospho-Ser404 were from Abcam (Cambridge, UK), and anti-phospho-Thr181, phospho-Thr205 and AT180 (P-T231) were from Invitrogen (Carlsbad, CA). Anti-actin was from Sigma (St. Louis, MO). The plasmid containing human tau with one N-terminal insertion and four microtubule-binding (MTB) repeats (1N4R) was used and is referred to here as the tau WT^59^. Plasmids encoding the following mutation in 1N4R human tau were also used: those containing single alanine substitutions (1A mutants) at Ser202 (S202A), Thr205 (T205A), Ser235 (S235A) or Ser404 (S404A); those containing three alanine substitutions (3A mutants) at Thr205, Ser235, and Ser404 (205A/235A/404A); at Ser202, Ser235, and Ser404 (202A/235A/404A); at Ser202, Thr205, and Ser404 (202A/205A/404A); and at Ser202, Thr205, and Ser235 (202A/205A/235A); and those containing four alanine substitutions at Ser202, Thr205, Ser235 and Ser404 (4A). The tau FTDP-17 mutant plasmids encoding R5H, R5L, K257T, G272V, P301L, P301S, V337M, E372G, G389R, R406W, N410H and T427M have been described elsewhere^18^,^59^.

### Preparation of mouse brain extracts

All animal experiments were performed according to the guidelines for animal experimentation of Tokyo Metropolitan University. The study was approved by the Research Ethics Committee of Tokyo Metropolitan University (approval number, A27-45). All efforts were made to reduce the suffering of animals used. The P301L tau transgenic mouse line (JNPL3) was housed in the animal facility of the Tokyo Metropolitan Institute of Medical Science, as described previously^16^. The cerebral cortices from mouse brains were homogenized in 10 mM Tris-HCl, pH 7.4, 1 mM EGTA, 0.8 M NaCl, 10% sucrose, 10 mM NaF, 10 mM β-glycerophosphate (Merck Millipore, Darmstadt, Germany), 0.4 mM 4-(2-aminoethyl)-benzenesulfonyl fluoride hydrochloride (Pefabloc) (Wako Chemicals, Osaka, Japan), 10 μg/ml leupeptin (Peptide Institute, Osaka, Japan), and 1 mM dithiothreitol. After centrifugation at 15,000 × *g* for 20 min, the supernatant was used as the brain extract.

### COS-7 cell culture and Preparation of Cell Extracts

COS-7 cells were maintained in Dulbecco’s modified Eagle’s medium (Sigma) supplemented with 10% (v/v) fetal bovine serum (Hyclone, GE Healthcare, Tokyo, Japan), 100 U/ml penicillin, and 0.1 mg/ml streptomycin. Cells were transfected with the indicated plasmids using HilyMax transfection regent (Dojindo, Kumamoto, Japan), according to the manufacturer’s protocol. After transfection for 24 h, the cells were lysed in 20 mM HEPES, pH 7.4, 1 mM MgCl_2_, 100 mM NaCl, 0.5% (v/v) Nonidet P-40, 0.4 mM Pefabloc, 10 mM NaF, 10 mM β-glycerophosphate, 10 μg/ml leupeptin and 1 mM dithiothreitol on ice for 10 min. After centrifugation at 15,000 × *g* for 15 min, each supernatant was retained.

### *In vitro* and metabolic phosphorylation

Cdk5-p25 was purified from Sf9 cells infected by baculovirus encoding Cdk5 and p25, as described previously^60^. Tau proteins were purified from the heat-treated extract of *Escherichia coli* BL21-CodonPlus (DE3)-RP using a Mono S column with an AKTA purifier (GE Healthcare)^19^. The amount of tau protein was estimated by Coomassie Brilliant Blue staining of gels using bovine serum albumin as a standard. Tau at 0.1 mg/ml was phosphorylated by Cdk5-p25 at 35 °C for 0 to 3 h in 10 mM MOPS, pH 6.8, 2 mM MgCl_2_, 0.1% Nonidet P-40, and 1 mM ATP.

COS-7 cells were labeled by incubation for 4 h with [^32^P]orthophosphate 24 h after transfection. The cell lysate was boiled in the presence of 0.5 M NaCl, and the heat-stable supernatant was obtained by centrifugation at 100,000 × g for 30 min. Tau was immunoprecipitated using Tau5 antibody, followed by binding to protein G Sepharose (GE Healthcare), as described previously^19^. Two-dimensional phospho-peptide map analysis was performed, as described previously^18^. The phosphorylation signal was captured by a FLA7000 bioimage analyzer (GE Healthcare) and the data were analyzed using Image J software.

### SDS-PAGE, Phos-tag SDS-PAGE and Immunoblotting

Laemmli’s SDS-PAGE was performed using 12.5% (w/v) polyacrylamide gels, and Phos-tag SDS-PAGE was performed using 7.5% (w/v) polyacrylamide gels containing 50 μM Phos-tag acrylamide (Wako Chemicals) and 150 μM MnCl_2_, as described previously^20^,^21^. For immunoblotting, the proteins separated in the gel were transferred to a PVDF membrane (Merck Millipore) using a submerged blotting apparatus and then visualized using Enhanced Chemiluminescence Detection kit reagents (GE Healthcare).

## Additional Information

**How to cite this article**: Kimura, T. *et al.* Quantitative and combinatory determination of *in situ* phosphorylation of tau and its FTDP-17 mutants. *Sci. Rep.* **6**, 33479; doi: 10.1038/srep33479 (2016).

## Supplementary Material

Supplementary Information
