# Supplementary material for: Quantitative and combinatory determination of in situ phosphorylation of tau and its FTDP-17 mutants
Source: Sci Rep. 2016 Sep 19;6:33479. doi: 10.1038/srep33479 (PMC5027580; doi:10.1038/srep33479)

**Quantitative and combinatory determination of *in situ* phosphorylation of tau and its FTDP-17 mutants**

**Taeko Kimura^1,*^, Tomohisa Hosokawa^1,‡^, Masato Taoka^2^, Koji Tsutsumi^1,‡^, Kanae Ando^1^, Koichi Ishiguro^3^, Masato Hosokawa^4^, Masato Hasegawa^4^, and Shin-ichi Hisanaga^1,*^**

^1^Laboratory of Molecular Neuroscience, Department of Biological Sciences, ^2^Department of Chemistry, Tokyo Metropolitan University, Hachioji, Tokyo 192-0397

^3^Juntendo University, Tokyo, Japan 113-0033

^4^Tokyo Metropolitan Institute of Medical Science, Setagaya, Tokyo 156-8506

‡Present address: Tomohisa Hosokawa at Brain Science Institute, Riken; Koji Tsutsumi at Faculty of Sciences, Kitasato Universi

*To whom correspondence should be addressed: Taeko Kimura and Shin-ichi Hisanaga, Department of Biological Sciences, Tokyo Metropolitan University, Hachioji, Tokyo 192-0397, Japan.

Tel: +81-42-677-2769, Fax: +81-42-677-2559

E-mail: kimura-taeko@tmu.ac.jp, hisanaga-shinichi@tmu.ac.jp

**Legends of Supplementary Figures.**

**Supplementary Figure 1.** The reactivity of anti-phospho-Ser202 (P-S202). Tau and its Ala mutant at Ser202 (S202A) or Thr205 (T205A) was expressed with (+) or without Cdk5-p35 (-) in COS-7 cells (upper) or incubated in vitro with Cdk5-p25 (+) or not (-) (lower), and then immunoblotted with anti-P-S202 (Abcam).

**Supplementary Figure 2.** Phosphorylation of Cdk5-sites in FTDP-17 tau with a mutation in the C-terminal region analyzed by immunoblotting with phospho-specific antibodies and 2D-phospho-peptide mapping. (A) Tau WT, G389, M410H or T427M mutants were co-expressed with Cdk5-p35 in COS-7 cells. Their phosphorylation at Ser202, Ser235 and Ser404 was detected by immunoblotting with phospho-specific antibodies. Total tau is shown by immunoblotting with Tau5. Their quantification is shown on the right (the mean ± SD, n=3). (B) 2D-phospho-peptide map of tau WT or its mutant phosphorylated by Cdk5-p35 in COS-7 cells. Tau WT, G389R, M410H or T427M was phosphorylated by Cdk5-p35 in the presence of [g-^32^P]ATP. Tau was prepared by immunoprecipitation and analyzed by a 2D-phosphopeptide map. Arrowheads indicate the Cdk5-phosphorylation spots.

**Supplementary Figure 3.** Uncropped examples of immunoblotting of tau, Cdk5, p35 and actin.


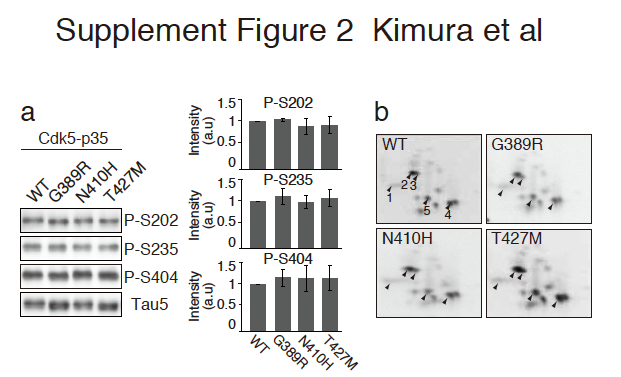

Supplement: Supplementary Information [file srep33479-s1.doc]
